# Artificial intelligence as a virtual coach in a cognitive behavioural intervention for perfectionism in young people: A randomised feasibility trial

**DOI:** 10.1016/j.invent.2024.100795

**Published:** 2024-11-30

**Authors:** Catherine Johnson, Sarah J. Egan, Per Carlbring, Roz Shafran, Tracey D. Wade

**Affiliations:** a Flinders University Institute of Mental Health and Wellbeing and Blackbird Institute, Adelaide, Australia; b enAble Institute, Faculty of Health Sciences, Curtin University, Perth, Australia; c Discipline of Psychology, School of Population Health, Curtin University, Perth, Australia; d Department of Psychology, Stockholm University, Sweden; e School of Psychology, Korea University, Seoul, South Korea; f Great Ormond Street Institute for Child Health, University College London, United Kingdom

**Keywords:** Perfectionism, Treatment, Artificial intelligence, Anxiety, Disordered eating, Guided self-help

## Abstract

**Background:**

We examined the feasibility and outcomes of Artificial Intelligence (AI) as a virtual coach in guided self-help (GSH-AI) compared to pure self-help (PSH).

**Method:**

Participants (*N* = 85 undergraduate university students; M age = 20.65 years [*SD* = 2.38]; 84 % female) were randomised to PSH (*N* = 42) or GSH-AI (*N* = 43). The intervention was a brief 11-module online cognitive behaviour therapy for perfectionism intervention completed over 4-weeks. GSH-AI participants were given suggested questions to ask AI for guidance in completing the intervention. Data were collected at baseline, 4- and 8-weeks post-randomisation.

**Results:**

Engagement was good, only one person in each group did not use any modules; module completion was equivalent across conditions (6.67, *SD* = 3.22 and 6.18, *SD* = 3.42 respectively). Between baseline and post-intervention people in the GSH-AI condition showed an almost 3.5 times increase in preferring support to be received from AI versus other modes of support. Only 52 % and 22 % of participants completed 4- and 8-week post-randomisation surveys, with no differences in psychological outcomes between the PSH and GSH-AI groups. Main effects of time indicated moderate to large within-group effect size improvements for disordered eating, stress, anxiety, and perfectionism.

**Conclusions:**

Qualitative feedback indicated that AI was initially acceptable as a guide and became even more acceptable after it had been experienced. Fully powered trials are required to determine the impact of AI guidance on outcomes, and whether type of AI platform (customised versus generic) and type of mental health disorder interact with its effects.

## Highlights

• We examined the feasibility of AI-guided internet CBT for perfectionism.
• Barriers included lack of AI familiarity and the impersonal nature of responses.
• Facilitators included ease of gaining valuable, confidential, tailored information.
• Preference for AI support increased strongly after experiencing it.
• The addition of generic AI support did not improve psychological outcomes.

A meta-analysis by Curran and Hill (2019) demonstrated a significant increase in perfectionism over a 27-year period. This occurred across different facets of perfectionism: self-oriented (excessively high personal standards), socially prescribed (perceiving social context to be demanding, that others judge them harshly, and that they are increasingly inclined to display perfection as a means of securing approval), and other oriented (imposing more demanding and unrealistic standards on others). While self-oriented perfectionism has been considered by some to be advantageous, evidence shows it to be associated with significantly poorer mental health outcomes, including symptoms of depression, anxiety, and eating disorders (Bills et al., 2023; Callaghan et al., 2024; Lunn et al., 2023; Stackpole et al., 2023), as well as academic burnout and procrastination (Osenk et al., 2020). This rise in perfectionism was documented in university students, a population who report lower levels of wellbeing than their age matched peers in the general population, which in turn is associated with increased risk of academic failure, lower GPA, withdrawal from university, and suicide (Bruffaerts et al., 2018; Thorley, 2017; Zając et al., 2024).

One approach to improve mental health is internet interventions. Cognitive behaviour therapy for perfectionism (ICBT-P) is an example of a transdiagnostic intervention that improves perfectionism and symptoms of depression, anxiety, and disordered eating (Galloway et al., 2022; Robinson and Wade, 2021). Maintaining participant engagement (i.e., use of interventions), however, is a well-recognised difficulty when delivering internet interventions for mental health (Lipschitz et al., 2023). Open trials are associated with poor uptake and controlled trials achieve between 50 % and 90 % completion rates (Musiat et al., 2022). Guidance, however, can significantly increase the average amount of intervention completion and the proportion of intervention completers (Musiat et al., 2022).

Guidance can be offered in many ways (Smoktunowicz et al., 2020) from brief weekly emails or telephone contact, SMS reminders, or even guidance on demand (the option to contact their clinician if needed; Käll et al., 2023). The advent of Artificial Intelligence (AI) has sparked discussion about its use as a method of guidance, with a call to “carefully study client preferences, the effects, and consequences” (p.2, Carlbring et al., 2023). Recently we engaged in a small pilot study of the feasibility and preferences of an international panel of young people with lived experience of anxiety or depression, who reported they would be interested in engaging in AI guided ICBT-P and helped to co-design the intervention which we aimed to examine evidence for feasibility, acceptance, and initial efficacy in the current study (Egan et al., 2024).

The aim of the current study was to examine feasibility of AI in a pilot randomised controlled trial comparing use of AI for guidance in the delivery of ICBT-P compared to no AI guidance in university students. Our overall aim was to examine feasibility of AI as a guide, as indicated by engagement and acceptability. Our primary hypotheses were that most participants would engage with AI for guidance during the intervention, and most participants would rate this as better than no support (i.e., pure self-help), as suggested in other studies such as Lopes et al. (2023). Our secondary hypotheses were that we would see greater module completion and larger improvements in psychological outcomes post-intervention in the AI guided group compared to pure self-help. Given evidence for perfectionism as both a transdiagnostic risk and maintenance factor across anxiety, depression and eating disorders (e.g., Egan et al., 2011), we have included these outcomes together with a more general measure of distress (i.e., stress) across our sample of young adults.

## 1 Method

### 1.1 Participants

Psychology students were recruited from the volunteer research pools at Flinders University (Adelaide, South Australia) and Curtin University (Perth, Western Australia) where participation earns course credit. Participants were required to be between 18 and 29 years of age, fluent in English, and self-identifying with experiencing perfectionism as a problem, based on the following lay description in recruitment materials: “Unhealthy perfectionism involves harsh self-criticism, fear of making mistakes, and/or basing your self-worth almost entirely on achievements; this is different from striving for excellence, which is a good thing.”. Past involvement with perfectionism treatment research was the sole exclusion criterion.

Longitudinal power analysis (Hedeker et al., 1999) showed that to detect a Cohen's *d* between-group effect size of 0.55 between two active interventions (Shu et al., 2019), with α = 0.05, power = 0.80, and 25 % attrition between baseline and one-month follow-up, 54 participants were required at baseline (27 in each intervention group).

### 1.2 Design

Students were randomised to either (1) guided self-help using AI assistance (GSH-AI) or (2) pure self-help (PSH) for a 4-week online perfectionism intervention that was delivered as an interactive PDF document. Primary outcome measures (use, usefulness, and acceptability of AI support) were measured at post-intervention except for type of support preferred, and current use of AI, which were also measured at baseline. Secondary outcome measures (stress, anxiety, depression, disordered eating, perfectionism) were measured at three time-points: baseline, post-intervention (4-weeks post-randomisation), and follow-up (8-weeks post-randomisation).

### 1.3 Procedure

Ethics approval was granted from the Human Research Ethics Committees of Flinders University (**ID:** 6460) and Curtin University (ID: HRE2024–0111). Upon completion of the baseline survey in Qualtrics, students were randomised within this platform to an intervention group, with a link then revealed for downloading the appropriate workbook (i.e., with or without instructions for using AI for support). Participants received an automated email generated within Qualtrics at 4- and 8-weeks with links to the post-intervention and follow-up surveys respectively. Due to slower than anticipated recruitment, requirement for the third timepoint included in the preregistration protocol was removed for the final 8 weeks of the 13-week study. It was not possible for participants or the researcher undertaking analyses (CJ) to be blind to the allocated treatment group (cf. Goldberg et al., 2023).

### 1.4 Intervention

All participants received an 11-module pdf workbook “*Changing Perfectionism”* to complete at their own pace over 4 weeks. Module topics included identifying perfectionism plus the pros and cons of perfectionism, plus experiments to challenge perfectionism and self-criticism, and are described in Table 1. Each module takes approximately 30 min to complete, in total 5.5 h for the entire intervention. The content is based on the book “Overcoming Perfectionism: A self-help guide using scientifically supported cognitive behavioural techniques” (Shafran et al., 2018), as summarised in Egan et al. (2012), and adapted from a manual tested with telephone guidance in Lowndes et al. (2018). The protocol is intended to be used flexibly, and has been found to be effective when used over an 8-week period (e.g., Rozental et al., 2024) or a over a 4-week period (e.g., Robinson et al., 2024).

**Table 1:** Content of the perfectionism intervention.

Module	Example questions to ask AI tool
Introduction to AI	“If you are not satisfied with the response, tell the AI tool an instruction like ‘I don't understand this, it is too complex’ or ‘Please re-generate this response’”
1. What is perfectionism?	NA
2. Why does it develop?	‘Tell me the reasons why perfectionism develops’
3. Identifying perfectionistic thoughts	‘I am meant to be monitoring my perfectionism thoughts, feelings and behaviours but it is really hard. Sometimes I forget, at other times I feel I am not doing it right. Can you suggest solutions to help me monitor my perfectionism in real time?’
4. What keeps perfectionism going?	‘I am trying to understand evidence-based ideas about what keeps my perfectionism going. Factors such as self-worth based on striving, evaluation of standards, avoidance, all-or-nothing thinking and self-criticism are all relevant. Can you help me understand how these fit together?’
5. Pros and cons of perfectionism	‘I need to work out the pros and cons of having perfectionism. I am not sure what they are for me personally. I am successful but also lack self-confidence. Are these my pros and cons?’
6. Challenging perfectionism myths	‘Can you explain the Yerkes-Dodson curve in relation to perfectionism please?’
7. Experiments to challenge perfectionism	‘I need to design a behavioural experiment to help me overcome perfectionism. What I want to try out is what happens if I go out with less make-up. I am afraid people might notice and comment negatively. Can you help me devise a behavioural experiment to test this please?’
8. Changing self-criticism	‘I forgot to save a document and had to re-do it. Although it didn't take that long, I keep on being very self-critical and unkind to myself in my head. That's also making me mad as it wasn't that big a deal and I know I shouldn't be so self-critical. I'm self-critical for being self-critical. Please help me stop being so self-critical’
9. Procrastination and pleasant events	‘I keep using ChatGPT to procrastinate doing my assignment, can you help me not to procrastinate?’ ‘Can you tell me a list of pleasant events to improve my mood?’
10. Self-evaluation	‘I need to decrease my self-worth being dependent on striving and achievement. I have been trying to use a pie chart, but I am not sure I am doing it right. Can you help?’
11. Planning for the future	‘I've done a lot of work and made progress on tackling perfectionism. What can I do to help make sure I don't relapse?’

Participants in the GSH-AI condition received a workbook with instructions on how best to use AI for guidance, together with sample questions to ask AI at the end of each module if further explanation or examples were required. Participants were free to use any AI tool, with Chat GPT offered as a suggestion for those unfamiliar with AI. Participants were also advised to *“ask for information about techniques or content, rather than personalised mental health advice, given AI tools are not able to diagnose and treat mental health difficulties like a person can”.*

The workbook was co-designed with young adults with lived experience of anxiety and depression, including participants from low- and middle-income countries (Egan et al., 2024) and is freely available to download (https://www.overcomingperfectionism.com/_files/ugd/4ae068_6762a10477e545f9a4b47c5b870673a0.pdf). Participants in the PSH condition received a workbook with these instructions removed and were specifically requested not to use AI to assist with the intervention.

### 1.5 Primary outcome measures: use, usefulness, and acceptability of AI support

Table 2 shows questions developed for this study assessing use of AI at baseline (item 1) and for support during the intervention (2), usefulness of AI support (3,6), and of workbook tips for utilising AI (4–5). Items 2–6 were only shown to participants in the GSH-AI group, at post-intervention. These participants were also asked what they liked most/least about using AI support (open ended question response box). At baseline, all participants were asked which type of support they would prefer for online interventions if they had a choice; this question only appeared for participants in the GSH-AI group at post-intervention. Completion rates were also measured across both intervention groups (self-reported number of modules completed).

**Table 2:** Use, usefulness, and acceptability of AI support: quantitative results.

Question (*N* respondents)	M *(SD)*	N *(%)*				
	x/5	Never	Rarely	Sometimes	Often	Always
1. How often do you currently use Artificial Intelligence (AI) Tools? (*N* = 82)^a^	2.35 *(1.04)*	19 *(23.2)*	29 *(35.4)*	21 *(25.6)*	12 *(14.6)*	1 *(1.2)*
2. Did you use Artificial Intelligence (AI) Tools for support with the perfectionism modules? (*N* = 22)	2.77 (*1.31)*	6 *(27.3)*	1 *(4.5)*	9 *(40.9)*	4 *(18.2)*	2 *(9.1)*

		Not at all useful	Slightly useful	Moderately useful	Very useful	Extremely useful
3. How useful did you find AI Tools as support during this intervention? (*N* = 19)	2.74 (*1.24*)	5 *(26.3)*	2 *(10.5)*	5 *(26.3)*	7 *(36.8)*	0
4. At the start of the workbook, we provided general tips on how to use AI Tools most effectively for support. How would you rate these instructions? (*N* = 19)	3.63 (*0.90)*	0	1 *(5.3)*	9 *(47.4)*	5 *(26.3)*	4 (*21.1)*
5. After each module, we provided sample questions for AI support. How would you rate these instructions? (*N* = 19)	3.58 (*0.84)*	0	2 *(10.5)*	6 *(31.6)*	9 *(47.4)*	2 *(10.5)*


Question (*N* respondents)	N *(%)*		
	Yes	No	Not sure
6. Were AI Tools better than no support (i.e., just reading the information)? (*N* = 19)	14 *(73.7)*	2 *(10.5)*	3 *(15.8)*

a This question was posed to the whole sample at baseline; remaining questions posed to AI-supported group only, at post-intervention.

### 1.6 Secondary outcome measures: psychological outcomes

To more sensitively detect change that may have occurred during the latter part of our four-week intervention period, we adjusted question time frames to reference “the past week” across all measures and timepoints (i.e., shortening clinical perfectionism, anxiety and disordered eating from the original 4-week scales; depression from 2-weeks).

#### 1.6.1 Stress

This was measured using the 7-item stress subscale of the Depression, Anxiety and Stress Scale (Short form; DASS-21, Lovibond and Lovibond, 1995). Items are scored from 0 (*never*) to 3 (*always*), with higher scores indicating greater distress over the past week. This measure has good psychometric properties in adult clinical (Brown et al., 1997) and non-clinical (Henry and Crawford, 2005) samples. Internal consistency in the current study was acceptable (McDonald's Omega; ꭥ) = 0.76.

#### 1.6.2 Anxiety

The 7-item Generalised Anxiety Disorder Scale (GAD-7; Spitzer et al., 2006) was utilised. Items are scored from 0 (*not at all*) to 3 (*nearly every day*); higher scores reflect greater anxiety. The scale has shown sound psychometric properties in an adult clinical sample (Spitzer et al., 2006); Ω for the current study was good = 0.82.

#### 1.6.3 Depression

This construct was measured using the 9-item depression module of the Patient Health Questionnaire (PHQ-9; Kroenke et al., 2001); scores range from 0 (*not at all*) to 3 (*nearly every day*) with higher scores indicating greater depression. This scale has demonstrated good reliability and validity in adult clinical and non-clinical populations (Kroenke et al., 2001); Ω for this study was acceptable = 0.76.

#### 1.6.4 Disordered eating

The 7-item Eating Disorder Examination Questionnaire – Brief Version (EDEQ-7; Grilo et al., 2015) was used. Items 1–3 are scored from 0 (*no days)* to 6 (*every day)* and items 4–7 are scored from 0 (*not at all)* to 6 (*markedly);* higher scores indicate greater risk*.* This measure has good internal consistency and validity in a university sample (Jenkins and Davey, 2020). For the current study, we applied the same Likert anchors *(no days – every day*) across all seven items; and internal consistency was excellent, Ω = 0.92.

#### 1.6.5 Perfectionism

Three measures were used to assess different elements of perfectionism. ***Concern over mistakes*** and ***Personal standards*** were measured using the 9-item and 7-item subscales respectively from the Frost Multidimensional Perfectionism Scale (Frost et al., 1990). Items are scored from 1 (*Strongly Disagree)* to 5 (*Strongly Agree)* such that higher scores indicate greater perfectionism*.* A timeframe of reference is not nominated for these items. These subscales have good internal consistency in university students (Franco et al., 2014). For the current study, internal consistency was good, Ω = 0.85 (Concern over mistakes) and 0.87 (Personal standards). ***Clinical perfectionism*** was measured via the 10-item version of the Clinical Perfectionism Questionnaire (CPQ), with acceptable psychometric properties shown in clinical and community samples (Egan et al., 2016). Items are scored from 1 (*Not at all*) to 4 (*All of the time*), with higher scores indicating greater perfectionism over the past week. A technical error in the online survey meant one section asked about symptoms over the last month, while another requested reporting over the previous week. This measure should therefore be interpreted with caution, as it may have confused participants and/or may not have detected changes that occurred during the latter part of the 4-week intervention. For the baseline survey, internal consistency was good, Ω = 0.82. In addition, a self-scoring version of the CPQ that could chart their progress weekly was supplied to each participant, given the evidence that feedback-informed interventions improve retention and outcomes (Delgadillo et al., 2018).

### 1.7 Statistical analysis

All statistical analyses were performed using IBM Statistical Package for the Social Sciences, Version 29.02. Logistic regression was used to test whether characteristics at baseline (age, gender, psychological outcomes) predicted absence at post-intervention or follow-up surveys. Qualitative feedback regarding AI use was analysed with reflexive thematic analysis, with themes generated inductively and coded semantically with an experiential orientation, following the six phases described by Braun and Clarke (2022): (1) Familiarisation with the dataset; (2) Coding; (3) Generation of initial themes; (4) Developing and reviewing themes; (5) Refining, defining and naming themes, and (6) Writing up. Steps were undertaken by CJ, then modified during Steps (4)–(5) in discussion with TDW. Module completion rates between groups were compared via independent *t-*tests. Repeated measures analyses for psychological outcomes were conducted via Linear Mixed modelling (LMM), enabling inclusion of cases with missing data via maximum likelihood estimation.

## 2 Results

### 2.1 Description of participants

**Fig. 1:**
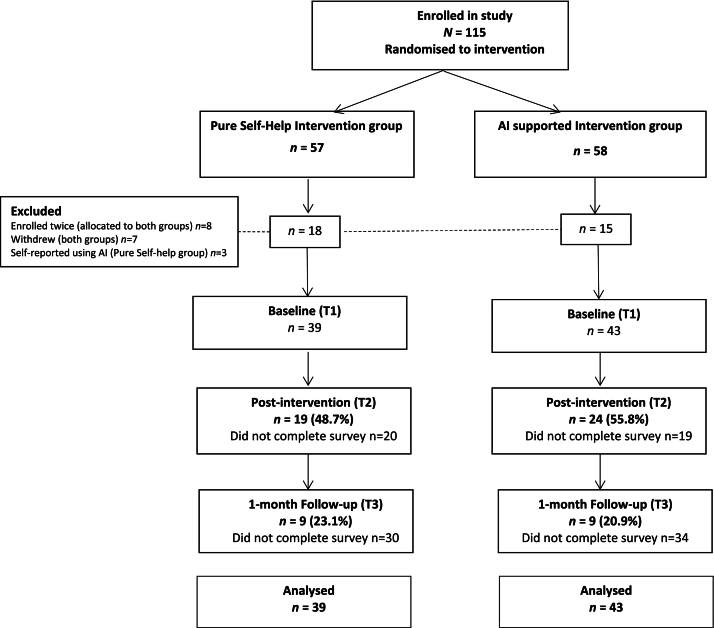
Flow of participants through study.

Fig. 1 shows the flow of participants through the study. Three participants in the PSH condition reported using AI for assistance during the intervention and were removed from analyses across timepoints. Of the 82 students who remained, mean age was 20.65 years (*SD* = 2.38), with mostly females (84.1 %) participating (12.2 % male, 3.7 % non-binary). Ethnicity was self-reported as follows: 70.7 % White, 12.2 % Mixed race, 11.0 % Asian, 3.7 % African, 1.2 % Aboriginal/Torres Strait Islander, 1.2 % Arab. Eighty three percent scored ≥29 on the Concern over mistakes subscale (Frost Multidimensional Perfectionism Scale), one standard deviation above published norms (Suddarth and Slaney, 2001), meeting the inclusion criteria of a previous ICBT-P study (Shafran et al., 2017).

### 2.2 Preliminary analyses

Attrition rates for the 4- and 8-week post-randomisation surveys were 52.3 % and 78 % respectively, much higher than the 25 % estimated in our a priori power analysis. Due to very small numbers, data from 8-weeks post-randomisation were not analysed. Independent t-tests were conducted to check baseline equivalence between randomised groups; these showed mean levels of *Concern over Mistakes* were higher in the PSH (*M* = 3.93, SD = 0.54) compared to the AI-GSH group (*M* = 3.59, SD = 0.74; *t*(80) = 2.38, *p* = .02). Given our analysis of interest was group*time (i.e., did one group change more than another over time?) rather than main effects of group, our analytic approach was not adapted. Logistic regression showed that treatment group, age, gender, and psychological measures at baseline did not predict completion of the post-intervention survey (Table 3), suggesting data were missing at random. All quantitative data were normally distributed. There were three (low scoring) outliers at baseline for perfectionism (concern over mistakes) which were retained in the analyses.

**Table 3:** Logistic regression analyses – predictors of absence at T2 (Post-intervention).

Predictor (baseline scores)	Absence at T2
	Odds ratio *(95* *% CI)*
Age	1.08 (0.89,1.30)
Gender	0.46 (0.14,1.54)
Treatment group	1.33 (0.56,3.17)

Psychological outcomes (baseline scores)	
Stress	0.78 (0.33,1.84)
Anxiety	1.09 (0.54,2.22)
Depression	1.41 (0.64,3.12)
Eating disorder risk	0.97 (0.77,1.24)
Perfectionism – concern over mistakes	1.18 (0.62,2.23)
Perfectionism – personal standards	1.35 (0.70,2.61)
Perfectionism – clinical	1.78 (0.82,3.86)

### 2.3 Use, usefulness, and acceptability of AI support

**Fig. 2:**
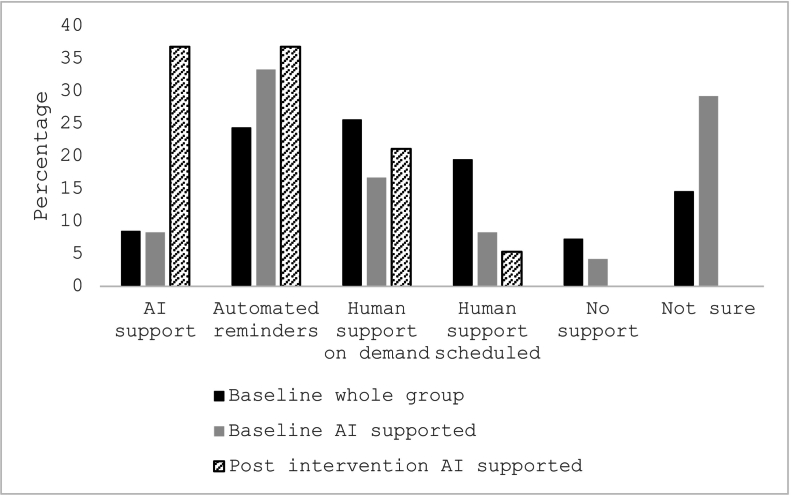
Preferred type of intervention support. Note. Post intervention, this question was only repeated for AI-GSH group.

Across the whole sample at baseline, 23.2 % of participants had never used AI tools, while 15.8 % used this technology *often* or *always* (Table 2). Preference was greatest for human support on demand (25.6 %; Fig. 2), or automated reminders (24.4 %), with few participants indicating a preference for support via AI (8.5 %).

The remaining results were obtained at post-intervention and pertain to the GSH-AI group. Here, preference for AI support increased from 8.3 % at baseline to 36.8 % at post intervention, following experience with using AI for guidance (Fig. 2). The increase largely came from participants who were not sure, or preferred no support, at baseline. During the intervention, about a quarter of participants used AI *often* or *always* to help, and a similar number never accessed this tool (Table 2). There were 63.1 % of participants who found AI support *moderately* or *very useful*, and 73.7 % reported AI tools were better than no support. There was very high support for the usefulness of the workbook tips for using AI (94.8 % *moderately* - *extremely useful*) and for the sample questions provided for AI support after each module (89.5 % *moderately* - *extremely useful*).

For most liked aspects of AI support, two key themes emerged from the 16 participants (66.7 % of the intervention group) who responded to this question. The first was “ease of use/efficiency”*.* Participants described how AI helped them apply the intervention to their situation (*n* = 6 e.g., “*I liked how if I asked for examples or personalised inputs it gave valuable responses that helped me understand my condition”*), on the ease of obtaining easy to understand answers (*n* = 5 e.g., *“Easy to get more information/explanation on the topic”*, *“It provided me with good, easy to read information”)*, accessibility (*n* = 4 e.g., *“I liked the freedom of asking questions anytime that I need it”*) and introduction to a new skill (*n* = 1, *“It allowed me to explore a new way of learning”*)*.* The second theme was “Personalised support”: describing the interactive, supportive, and confidential nature of AI (n = 4 e.g., “*It was interactive and felt like it wasn't all up to me”).*

Least liked aspects, answered by 17 respondents (70.8 %), grouped into almost directly opposing themes. The first was “difficulty/unfamiliarity”. Subthemes included needing to ask very specific questions (n = 5 e.g., *“it is only as good as the prompts the user gives AI”*), general unfamiliarity with AI (*n* = 3 e.g., *“it felt strange”; “Suggest more guided questions to ask AI tools in different scenarios”)* and feeling like AI did what they personally should be attempting *(n* = 2 e.g., *“felt like cheating”*). Participants also reported some negative feedback about AI including that it “*made things more complicated*”, and “*I do not trust the information provided and think it could be a bunch of nonsense*”. The second theme that emerged was “Impersonal”. Within this theme, participants described the information generated by AI as very generic, with limited ability to understand emotions (n = 6 e.g., “*it wasn't really personal”* and *“[Suggest instead to] use the premium function of Chat AI, which is like talking to somebody”).*

Measured via self-report post-intervention, type of support did not impact the average number of modules completed (PSH *M* = 6.67 modules, *SD* = 3.22; GSH-AI *M* = 6.18 modules, *SD* = 3.42; Cohen's *d* = 0.15, 95 % CI -0.48, 0.77), percentages completing more than half the modules (PSH 55.6 %; GSH-AI 54.5 %; *φ* = −0.01, *p* = .95), or engagement rates (one participant in each condition did not complete any modules).

### 2.4 Psychological outcomes

Descriptive statistics appear in Table 4. Comparison of scores between baseline and 4-weeks post-randomisation showed no significant group-time interactions (Table 5**)**. There were significant main effects of time with moderate to large within-group effect sizes for all outcomes except depression and disordered eating – that is, the perfectionism intervention improved these outcomes regardless of type of support. There were no significant main effects of group.

**Table 4:** Descriptive Statistics at Baseline (T1), 4- (T2) and 8-weeks (T3) Post Baseline for the Whole Sample, AI Guided, and Pure Self-help Groups.

Outcome variable		Whole group	AI guided (*N* = 43)^a^	Pure Self-help (*N* = 39)^a^
		Mean *(SD)*		
Stress	T1	1.67 (0.50)	1.61 (0.59)	1.74 (0.37)
	T2	1.28 (0.63)	1.30 (0.63)	1.26 (0.64)
	T3	1.18 (0.52	1.16 (0.64)	1.21 (0.42)
Anxiety	T1	1.62 (0.61)	1.54 (0.70)	1.71 (0.50)
	T2	1.25 (0.74)	1.21 (0.67)	1.31 (0.84)
	T3	1.25 (0.65)	1.27 (0.71)	1.23 (0.62)
Depression	T1	1.26 (0.55)	1.21 (0.55)	1.32 (0.55)
	T2	1.15 (0.83)	1.09 (0.81)	1.22 (0.86)
	T3	1.01 (0.59)	0.88 (0.62)	1.14 (0.56)
Eating disorder risk	T1	2.56 (1.77)	2.89 (1.89)	2.20 (1.58)
	T2	2.37 (1.67)	2.24 (1.68)	2.53 (1.69)
	T3	1.88 (1.95)	1.73 (1.56)	2.03 (2.36)
Clinical perfectionism	T1	2.61 (0.58)	2.60 (0.59)	2.63 (0.57)
	T2	2.20 (0.60)	2.13 (0.64)	2.28 (0.56)
	T3	2.18 (0.68)	2.33 (0.84)	2.03 (0.49)
Concern over mistakes	T1	3.75 (0.67)	3.59 (0.74)	3.93 (0.54)
	T2	3.20 (0.87)	3.03 (0.89)	3.40 (0.84)
	T3	3.19 (1.05)	3.17 (1.17)	3.20 (0.98)
Personal standards	T1	3.95 (0.67)	3.88 (0.68)	4.04 (0.66)
	T2	3.55 (0.73)	3.34 (0.68)	3.80 (0.74)
	T3	3.65 (0.70)	3.75 (0.81)	3.56 (0.60)

a Numbers at T1; at T2 *N* = 24/19 (AI supported/Pure Self-help); at T3 *N* = 9/9.

**Table 5:** Results of Linear Mixed Model Analysis: Time (Baseline, Post-intervention) by Group (AI Guided vs Pure Self-help).

Outcome measures	Main effect of time ES (95 % CIs)^a^, ^b^, ^d^	Group*time ES (95 % CIs)^a^, ^c^, ^e^
Stress	**−0.74 (−1.12, −0.36)**	0.45 (−0.09, 0.99)
Anxiety	**−0.60 (−0.98, −0.22)**	0.14 (−0.38, 0.66)
Depression	−0.26 (−0.63, 0.11)	0.06 (−0.51, 0.63)
Eating disorder risk	−0.12 (−0.49, 0.25)	−0.28 (−0.79, 0.23)
Clinical perfectionism	**−0.82 (−1.20, −0.44)**	−0.16 (−0.75, 0.42)
Concern over mistakes	**−0.84 (−1.23, −0.46)**	0.14 (−0.46, 0.74)
Personal standards	**−0.71 (−1.09, −0.33)**	−0.13 (−0.58, 0.32)

a ES = effect size (Cohen's *d*) using EMM/SE.

b Within-group (calculation using canonical form).

c Between-group, adjusted for baseline differences in outcome.

d Negative effect size shows improvement.

e Negative effect size favours AI; bold indicates significant effect.

## 3 Discussion

The primary outcome of the current investigation was the feasibility of using Artificial Intelligence (AI) in guided self-help (GSH-AI) in young people aged 19–29 years who self-reported perfectionism as being problematic. We found most participants were willing to engage with AI support during the guided intervention. While 68 % of the participants in the GSH-AI condition used AI sometimes to always during the intervention, around one third used it never or rarely. Potential hypotheses to explain the third of participants who did not use AI include possible concerns over AI accuracy, confidentiality, or a desire to work independently, which should be investigated in future research. However, experience in using AI for support resulted in an increase in preference for AI guidance by post-intervention (8.3 % to 36.8 %). AI support may become preferable as AI usage increases generally across the population when an individual discovers it can be a helpful adjunct for self-help treatment. Engaging less familiar or more reluctant groups, however, may continue to require novel strategies or initial demonstrations to help overcome initial concerns. This finding aligns with the results from Jain et al. (2024), which highlight how awareness of AI involvement can significantly alter user perceptions of the interactions, with human responses generally perceived as more authentic and practical compared to AI responses . This emphasizes the importance of understanding the role of user expectations and trust in the effectiveness of AI-supported interventions in mental health.

We also found that most participants reported AI tools were better than no support (74 %). Reported advantages of AI support included ease of use, efficiency with the intervention, accessibility of support at any time, and personalised, confidential support, in line with previous research (Egan et al., 2024). Disadvantages mirrored the same themes, with some participants citing the impersonal nature of support, and the need for very specific prompts to use the tool effectively, also like our previous research (Egan et al., 2024). Although there was high support for the workbook tips and sample questions, some participants may benefit from even greater explanations regarding the most effective ways to harness AI tools. Adding descriptions of the unique benefits such as tailored, 24-h support may boost numbers who utilise AI when it is offered.

Our secondary aim was to examine any differences in outcome between our AI-assisted and PSH groups. We predicted that AI-support would improve completion rates and outcomes. Neither hypothesis was supported. Module completion rates were moderate (55 %) and did not differ by type of support and were almost identical to previous studies testing unguided versions of ICBT-P in adolescent and university samples (Shu et al., 2019; Wade et al., 2019). There were no differences in outcomes between the groups. An effect may emerge in a larger sample; however, this remains to be tested. Across both groups combined, significant moderate (stress, anxiety) to large (clinical perfectionism, concern over mistakes, personal standards) improvements were shown for five of seven outcomes at post-intervention, demonstrating once again the effectiveness of ICBT-P (Galloway et al., 2022; Grieve et al., 2022; Robinson and Wade, 2021; Shu et al., 2019; Wade et al., 2019).

One explanation for the lack of effect relates to our use of AI as a tool that was not integrated into the intervention. This design was intentional, with our study assessing the potential of freely available, general AI platforms, supporting scalability for populations where access to bespoke and updated solutions might be limited. Further, embedded AI tools, which rely on custom-built, decision tree responses, may reduce the capacity for fully agile, conversational responses. However, the reliance on users to independently initiate interaction with AI might have presented a barrier, especially for participants unfamiliar with leveraging AI for guidance. While current general AI platforms have evolved to adopt an empathetic conversational tone, we recognize the potential for more integrated systems to boost outcomes by fostering a “technological alliance” (Goldberg et al., 2024), through incorporating elements such as regular, personalised check-ins and reminders to engage, dynamic feedback tailored to individual progress, and mood-based prompts. Embedding principles from frameworks such as the Virtual Therapist Alliance Scale (VTAS; Miloff et al., 2020) may help foster trust and a sense of partnership that simulates a collaborative therapeutic relationship. Moving forward, both forms of AI (integrated/custom built or freely available/general platforms) may have a place, and future research should continue to explore and compare these approaches in differing contexts.

Another potential explanation for our lack of effect is that type of mental health disorder might interact with the uptake and impact of AI guidance. Our co-design pilot study utilised a sample with lived experience of anxiety and depression; it may be that individuals with depression derive greater benefit from a tool that generates ideas, compared to our perfectionistic cohort who may be driven to complete the intervention regardless of access to support. Although our feasibility study was not adequately powered to explore this, different types of perfectionism (not measured in the current study) might also interact uniquely with the use of AI. For example, elevated socially prescribed perfectionism may inhibit people from using AI as they believe it may be interpreted as a sign of their own personal deficiency and inadequacy. It may also be that AI poses an extra burden of doing something “right” in a group with elevated levels of self-oriented perfectionism, and they accordingly elect to avoid its use as they fear not using it competently. Future qualitative research may assist in better understanding how best to facilitate engagement with AI tools across and within different mental health disorders.

Our primary limitation relates to the high attrition rates from post-intervention surveys; although equivalent across groups and not related to baseline measures, this meant our study was underpowered. Further reducing power in our small sample, three participants (7 %) from the pure GSH group reported using AI for support and were removed from analyses to prevent contamination. The participant information sheet clearly described the two randomised conditions (i.e., pure GSH group not to access AI for workbook support, although invited to use as normal for any other purpose) together with a reminder to these participants immediately post-randomisation. One explanation for these participants using AI may be a habitual reliance on this tool to generate ideas or seek clarification: all three reported prior use (ranging from rarely-often). It may be that as use of this tool becomes widespread in the community, it becomes harder to cleanly test interventions with and without such support.

Our second group of limitations relate to the type of AI intervention utilised in our study as follows: (a) We did not measure which AI tool was used by those in the AI-GSH group. Our participants were invited to utilise any AI tool should there be one they preferred, as well as providing suggestions (e.g., ChatGPT) for those who were not current users. In a larger sample, this will be useful to measure as a potential moderator of impact; (b) The use of structured prompts in our intervention could be seen as inadvertently standardising the AI-user interaction and limiting the AI's ability to provide a genuinely adaptive experience. The prompts provided in the accompanying booklet were intended as optional starting points to reduce barriers to engagement, particularly for participants unfamiliar with using AI. Importantly, these prompts did not prescribe the full course of the interaction, leaving substantial flexibility for participants to explore diverse topics and conversational pathways; (c) The potential for unreliability of information provided by a general AI platform may have impacted the study's outcomes and/or patient trust. By contrast, custom-built AI systems are able to incorporate domain-specific knowledge and decision-making frameworks, mitigating the risk of inaccurate responses. To counter this, our participant instructions for prompts included ‘evidence-based’ when searching for information using AI, as in our pilot testing this increased the accuracy of responses; the workbook also provided evidence-based content independent of AI-generated guidance; (d) While our participants were instructed to ask AI for information about technique or content rather than personalised mental health advice, and our suggested AI platform (ChatGPT) allows users to control whether their content is used to train AI, the flexibility in allowing participants to select their preferred AI platform introduces variability in data privacy and security measures, as these depend on the specific policies of the chosen tools. We recommend future research with general AI platforms specifically highlights tools with this privacy feature to ensure uniformity and enhanced security; (e) We note that, while AI was positioned as a tool to facilitate self-guided CBT, its use should not be misconstrued as equivalent to professional mental health advice where there is unique capacity to provide nuanced, clinical judgment or adapt dynamically to complex psychological needs. As highlighted in the participant intervention booklet, this underscores the importance of viewing AI as an adjunct to, rather than a substitute for, traditional therapeutic approaches.

A final limitation relates to the potential generalisability of our results to other populations. While our population of university students is very relevant given the elevated level of perfectionism and lower wellbeing in this group, our sample were psychology students receiving course credits. We also excluded those who had previous experience with perfectionism training. Evaluation of the intervention across a broader range of populations is warranted.

## 4 Conclusion

In a perfectionistic sample with low baseline usage of AI, lack of familiarity with the most effective way to use this tool was the most cited barrier, together with the impersonal nature of responses. Preference for AI support increased strongly by post-intervention after experiencing this approach, and most participants rated AI support as better than no guidance. Most liked elements included ease of gaining valuable, confidential, tailored information. AI support compared to pure self-help did not improve completion rates or psychological outcomes, with our sample underpowered due to high attrition. Future research should continue to examine, in larger samples, whether AI is effective as a potentially scalable solution to provide guidance in internet psychological interventions without the need for human input, whether impact is amplified by incorporating elements that mimic human alliance via custom-built AI platforms, and whether the type of mental health disorder interacts with this form of guidance.

## Funding sources

No funding was received for this project.

## Declaration of competing interest

Egan, Shafran and Wade receive royalties for the book “Overcoming Perfectionism: A self-help guide using scientifically supported cognitive behavioural techniques” which informs the content of the intervention.

## Data Availability

Data are available at reasonable request.
